# The association between skinfold thicknesses and estimated glomerular filtration rate in adolescents: a cross-sectional study

**DOI:** 10.1186/s12882-022-02709-7

**Published:** 2022-03-05

**Authors:** Yongchang Yang, Yubin Wu

**Affiliations:** Department of Pediatrics, Shengjing Hospital of China Medical University, Shenyang, China

**Keywords:** Triceps skinfold thickness, Subscapular skinfold thickness, Estimated glomerular filtration rate, NHANES, Cross-sectional study

## Abstract

**Background:**

Obesity is one of the causes of glomerular hyperfiltration. Studies on the relationship between body fat content and glomerular hyperfiltration have been limited to special children. Therefore, we aimed to evaluate the correlation between skinfold thickness, which represents body fat content, and estimated glomerular filtration rate (eGFR).

**Methods:**

The cross-sectional study included 6655 participants (3532 boys and 3123 girls; age: 12 − 17.99 years); data was obtained from the National Health and Nutrition Examination Survey (NHANES; 2001–2010). The independent variables were subscapular skinfold thickness and triceps skinfold thickness. The dependent variable was eGFR. We used multivariate linear regression models to evaluate their associations and also performed subgroup analyses.

**Results:**

After adjusting for age, standing height, race, family income, blood urea nitrogen and uric acid variables, multivariate regression analysis identified that triceps skinfold thickness and subscapular skinfold thickness were positively correlated with eGFR and glomerular hyperfiltration in boys. In subgroup analyses stratified by age and body mass index, triceps skinfold thickness was also associated with glomerular hyperfiltration in boys. There was a linear relationship between triceps skinfold thickness and eGFR in boys (β = 0.389, *P* < 0.001) and girls (β = 0.159, *P* = 0.0003).

**Conclusions:**

Triceps skinfold thickness and subscapular skinfold thickness are positively correlated with eGFR and glomerular hyperfiltration in US male adolescents. In all adolescents, there is a linear relationship between triceps skinfold thickness and eGFR.

**Supplementary Information:**

The online version contains supplementary material available at 10.1186/s12882-022-02709-7.

## Background

Obesity is an independent risk factor for chronic kidney disease [1], resulting in metabolic abnormalities as well as hemodynamic and renal structural changes. These decompensated mechanisms lead to obesity-related glomerular diseases. Histologically, obesity-related glomerular diseases are characterized by glomerular hypertrophy and focal segmental glomerulosclerosis [2]. One of the early signs of obesity-related glomerular diseases is the increase of glomerular filtration rate (GFR). Studies have shown that obesity [3] is one of the causes of glomerular hyperfiltration in adults [4] and adolescents [5]. The pathologic process of glomerular hyperfiltration is not fully understood, but it is well known that glomerular hyperfiltration eventually leads to podocyte detachment, proteinuria, and the development of chronic kidney disease [1]. In addition, glomerular hyperfiltration was found to be an independent risk factor for all-cause mortality in healthy adults [6].

Although skinfold thickness is not the gold standard for the diagnosis of obesity, it is a more accurate measurement of body fat content than body mass index (BMI) [7]. Skinfold thickness has been shown to be associated with excess fat in obese and non-obese adolescents [7]. Although the measurement error is large, skinfold thickness is relatively easy to obtain [8], so it is widely used in the evaluation of obesity and fat content in children and adolescents [9]. This body measurement method can better identify excess fat in an adolescents body [10]. In addition, skinfold thickness has been shown to be closely related to other nutritional and biochemical indicators [7].

Current, research on glomerular hyperfiltration rates in adolescents is mostly limited to special children [11]. Consequently, there is no consensus on the definition of glomerular hyperfiltration in adolescents as there is for adults [3]. The range of glomerular hyperfiltration in adults is reportedly 130-140 mL/min/1.73m^2^ [12]. The relationship between excess body fat and GFR in normal adolescents remains to be studied. Therefore, the purpose of this study was to investigate whether skinfold thickness is related to glomerular hyperfiltration in adolescents.

## Methods

### Study population

All data for this study were obtained from the National Health and Nutrition Examination Survey (NHANES), an ongoing duplicate cross-sectional study designed to assess the lifestyle, health, and nutritional status of the non-institutionalized civilian population of the United States. The survey consists of interviews conducted in participants’ homes and standardized physical examinations, including a blood sample, conducted in mobile examination centers. The data in this study were selected from five study intervals within NHANES that occurred between 2001 to 2010.

### Data collection

TSFT, SSFT, standing height and body weight were measured by trained technicians using standard procedures recommended by NHANES [13]. The Holtain pleated caliper (Holtain Ltd, Crymych, UK) was used to measure the skinfold thickness at the triceps and subscapular region accurately to 0.1 mm.

The serum creatinine (Scr) concentration was determined by the isotope dilution mass spectrometry (IDMS) standardized method starting in 2008 [14]. Before 2008, Scr concentration was measured using a modified Jaffe kinetic method that required no correction compared to IDMS [15]. eGFR was calculated using the Schwartz equation, derived from the Chronic Kidney Disease in Children study: eGFR(mL/min/1.73m^2^) = 0.413 × height(cm)/Scr(mg/dL) [16].

There is no clear definition of adolescent glomerular hyperfiltration [3]. Considering the definitions used in previous studies, we defined glomerular hyperfiltration [5] as eGFR greater than the 95th percentile for sex and age among all specimens; eGFR was converted to a dichotomous variable according to the glomerular hyperfiltration definition.

The ethics review board of the National Center for Health Statistics approved all NHANES protocols; and guardians of all participants gave written informed consent for the use of data for this research [17].

### Statistical methods

Continuous variables such as age and all measurements obtained (including eGFR) were presented as mean ± standard deviation (SD). The categorical variables were presented as percentage or frequency values. Student’s t-test was used to compare two means, whereas the one-way analysis of variance (ANOVA) was used to compare multiple means. Pearson’s Chi-squared or Fisher’s exact tests were used to compare categorical variables.

A univariate linear regression model was used to assess the associations between clinical variables and eGFR. The relationship of both TSFT and SSFT with eGFR was estimated by calculating an odds ratio (OR) and 95% confidence interval (CI) using a multivariate regression model. The results from unadjusted, minimally adjusted, and fully adjusted analyses were shown simultaneously according to the recommendation of the The Strengthening the Reporting of Observational Studies in Epidemiology (STROBE) statement [18]. Covariates were included as potential confounders in the models if they changed the estimates of skinfold thickness on eGFR by more than 10%. We constructed three models: (1) unadjusted; (2) minimally adjusted model (adjusted for age, standing height and race); and (3) fully adjusted model (adjusted for age, standing height, race, family income, blood urea nitrogen and uric acid variables). Hierarchical multivariate regression analysis was used for age and BMI subgroup analyses. We further analyzed the linear relationship between TSFT and eGFR using the Generalized Additive Model (GAM) and by fitting smoothing curves (penalty spline method).

All analyses were performed using the statistical software packages R (http://www.R-project.org, The R Foundation) and EmpowerStats (http://www.empowerstats.com, X&Y Solutions, Inc., Boston, MA). The level of significance of each test was set at *P* < 0.05.

## Results

The study sample initially included 8,498 adolescents aged from 12 to 17.99 years. Of these, we excluded 1843 participants—303 due to height data missing, 833 due to serum creatinine data missing, 302 due to missing triceps skinfold thickness (TSFT), 318 due to missing subscapular skinfold thickness (SSFT), 85 pregnant girls and one adolescent each with estimated glomerular filtration rates (eGFR) at maximum and minimum that may have been incorrect data. The final sample included 6655 adolescents (3532 boys and 3123 girls). There was no significant difference in BMI between the two groups. Descriptive data for participants according to whether or not glomerular hyperfiltration occurred, expressed as means and standard deviations, are presented in Table 1. There were no significant differences in body weight, height and BMI between adolescents with and without glomerular hyperfiltration. The TSFT and SSFT of adolescents with glomerular hyperfiltration were significantly higher than those of adolescents without (*P* < 0.001; *P* = 0.008 respectively).

**Table 1:** Baseline characteristics of the total cohort and stratified according to glomerular hyperfiltration

Glomerular hyperfiltration	No (*n* = 6319)	Yes (*n* = 336)	*P*-value
Age (years)	14.964 ± 1.982	14.964 ± 1.985	0.999
Weight (kg)	62.957 ± 16.311	62.727 ± 20.124	0.804
standing height (cm)	165.148 ± 10.145	164.471 ± 10.836	0.235
BMI (kg/m^2^)	22.892 ± 4.735	22.878 ± 5.830	0.960
Creatinine (mg/dl)	0.754 ± 0.161	0.516 ± 0.097	< 0.001
uric acid (mg/dl)	4.986 ± 1.218	4.587 ± 1.146	< 0.001
eGFR (ml/min/1.73m^2^)	93.747 ± 16.550	135.058 ± 19.003	< 0.001
TSFT (mm)	15.470 ± 7.432	16.986 ± 7.657	< 0.001
SSFT (mm)	13.850 ± 7.159	14.918 ± 7.865	0.008
BMI (kg/m^2^)			0.014
< 20	1886 (29.870%)	122 (36.310%)	
> = 20, < 30	3849 (60.960%)	178 (52.976%)	
> = 30	579 (9.170%)	36 (10.714%)	

The TSFT and SSFT of adolescents with glomerular hyperfiltration were significantly higher than those of adolescents without (*P* < 0.001; *P* = 0.008 respectively)

*BMI* body mass index, *eGFR* estimated glomerular filtration rate, *BUN* blood urea nitrogen, *TSFT* triceps skinfold thickness, *SSFT* subscapular skinfold thickness

The association between skinfold thickness and eGFR varied between sexes. Therefore, gender stratification analysis was performed. The univariate analysis (Table 2) indicated that age, weight, and BMI were negatively correlated with eGFR for both boys and girls (*P* < 0.001). In boys, TSFT was positively correlated with eGFR (*P* < 0.001), and waist circumference was negatively correlated with eGFR (*P* < 0.001), but not in girls.

**Table 2:** The results of univariate analysis of correlation between variables and eGFR

	Effect size β(95%CI), *P*-value	
	Male	Female
Age (years)	-5.242 (-5.501, -4.983), < 0.00001	-2.777 (-3.079, -2.476), < 0.00001
Weight (kg)	-0.266 (-0.299, -0.233), < 0.00001	-0.126 (-0.172, -0.080), < 0.00001
standing height (cm)	-0.586 (-0.641, -0.532), < 0.00001	-0.242 (-0.332, -0.151), < 0.00001
BMI (kg/m^2^)	-0.589 (-0.713, -0.465), < 0.00001	-0.289 (-0.424, -0.155), 0.00003
TSFT (mm)	0.440 (0.354, 0.526), < 0.00001	-0.017 (-0.109, 0.076), 0.72339
SSFT (mm)	-0.016 (-0.106, 0.074), 0.73191	-0.011 (-0.098, 0.076), 0.80739
waist circumference (cm)	-0.092 (-0.139, -0.046), 0.00009	0.021 (-0.034, 0.076), 0.45502

The univariate analysis indicated that age, weight, and BMI were negatively correlated with eGFR for both boys and girls (*P* < 0.0001). TSFT was positively correlated with eGFR in boys (*P* < 0.0001), but not in girls

*BMI* body mass index, *eGFR* estimated glomerular filtration rate, *TSFT* triceps skinfold thickness, *SSFT* subscapular skinfold thickness

In minimally and fully adjusted models, multiple regression showed TSFT and SSFT were positively correlated with eGFR in boys. In girls, TSFT and SSFT were only positively correlated with eGFR in the fully adjusted model (Table 3). Based on multivariate regression analysis, we found that TSFT and SSFT were the only potential risk factors for glomerular hyperfiltration in boys (Table 4).

**Table 3:** Multivariate regression analysis of the correlations between the eGFR and skinfold by gender

Exposure	Effect size β (95%CI), *P*-value		
	Crude model	Minimally adjusted model	Fully adjusted model
Male			
TSFT (mm)	0.440 (0.354, 0.526) < 0.00001	0.248 (0.176, 0.320) < 0.00001	0.389 (0.318, 0.460) < 0.00001
SSFT (mm)	-0.016 (-0.106, 0.074) 0.73191	0.145 (0.070, 0.220) 0.00014	0.323 (0.247, 0.398) < 0.00001
Female			
TSFT (mm)	-0.017 (-0.109, 0.076) 0.72339	0.029 (-0.056, 0.115) 0.50353	0.159 (0.073, 0.244) 0.00027
SSFT (mm)	-0.011 (-0.098, 0.076) 0.80739	0.069 (-0.011, 0.150) 0.09179	0.208 (0.126, 0.290) < 0.00001
Total			
TSFT (mm)	0.233 (0.170, 0.296) < 0.00001	0.191 (0.135, 0.246) < 0.00001	0.322 (0.267, 0.376) < 0.00001
SSFT (mm)	-0.013 (-0.076, 0.049) 0.67773	0.118 (0.063, 0.174) 0.00003	0.285 (0.229, 0.340) < 0.00001

In minimally and fully adjusted models, multiple regression showed TSFT and SSFT were positively correlated with eGFR in boys; but in female, TSFT and SSFT were only positively correlated with eGFR in fully adjusted model

*TSFT* triceps skinfold thickness, *SSFT* subscapular skinfold thickness

Note: Crude model: We did not adjust any covariants. Minimally adjusted model: We adjusted age, race, and standing height. Fully adjusted model: We adjusted age, standing height, race, family income, blood urea nitrogen and uric acid variables

**Table 4:** Multivariate regression analysis of the correlations between the glomerular hyperfiltration and skinfold by gender

Exposure	Effect size OR (95%CI), *P*-value		
	Crude model	Minimally adjusted model	Fully adjusted model
Male			
TSFT (mm)	1.046 (1.027, 1.066) < 0.00001	1.043 (1.023, 1.064) 0.00002	1.065 (1.044, 1.087) < 0.00001
SSFT (mm)	1.029 (1.009, 1.050) 0.00379	1.026 (1.005, 1.047) 0.01466	1.051 (1.028, 1.074) 0.00001
Female			
TSFT (mm)	1.006 (0.983, 1.030) 0.61541	1.002 (0.977, 1.027) 0.90162	1.018 (0.991, 1.046) 0.19411
SSFT (mm)	1.010 (0.989, 1.032) 0.34360	1.006 (0.984, 1.029) 0.60648	1.021 (0.996, 1.047) 0.10792
Total			
TSFT (mm)	1.030 (1.015, 1.045) 0.00009	1.026 (1.010, 1.041) 0.00111	1.043 (1.027, 1.060) < 0.00001
SSFT (mm)	1.020 (1.006, 1.035) 0.00640	1.016 (1.001, 1.032) 0.03670	1.036 (1.019, 1.053) 0.00003

After stratification by gender, multiple regression analysis showed that TSFT and SSFT were risk factors for glomerular hyperfiltration only in boys

*TSFT* triceps skinfold thickness, *SSFT* subscapular skinfold thickness

Note: Crude model: We did not adjust any covariants. Minimally adjusted model: We adjusted age, race, and standing height. Fully adjusted model: We adjusted age, standing height, race, family income, blood urea nitrogen and uric acid variables

The results of a subgroup analysis stratified by age and BMI in boys are shown in Table 5. TSFT and SSFT were positively associated with glomerular hyperfiltration in boys. SSFT and TSFT were not associated with glomerular hyperfiltration in girls (data not shown). Stratified by BMI, TSFT and SSFT were positively associated with glomerular hyperfiltration in boys.

**Table 5:** Correlations between the glomerular hyperfiltration and triceps skinfold, subscapular skinfold by different age and BMI in boys

Exposure	Effect size OR (95%CI), *P*-value	
	TSFT	SSFT
Age (years)		
< 13	1.069 (1.005, 1.137) 0.0349	1.114 (1.044, 1.188) 0.0010
> = 13, < 16	1.052 (1.018, 1.088) 0.0025	1.058 (1.019, 1.098) 0.0029
> = 16, < 17.99	1.077 (1.044, 1.112) < 0.0001	1.035 (1.000, 1.071) 0.0518
BMI (kg/m^2^)		
< 20	1.109 (1.013, 1.213) 0.0244	1.166 (1.018, 1.334) 0.0262
> = 20, < 30	1.087 (1.047, 1.128) < 0.0001	1.048 (1.008, 1.089) 0.0178
> = 30	1.125 (1.035, 1.224) 0.0056	1.114 (1.020, 1.217) 0.0162

Stratified by age, TSFT was positively associated with glomerular hyperfiltration in male adolescents aged 12–17.99 years. Stratified by BMI, TSFT and SSFT were positively associated with glomerular hyperfiltration in boys

Abbreviations: TSFT, triceps skinfold thickness; SSFT, subscapular skinfold thickness

Note 1: Above are fully adjusted model: adjusted for age, standing height, race, family income, blood urea nitrogen and uric acid

Note 2: In each case, the model is not adjusted for the stratification variable itself

**Fig. 1:**
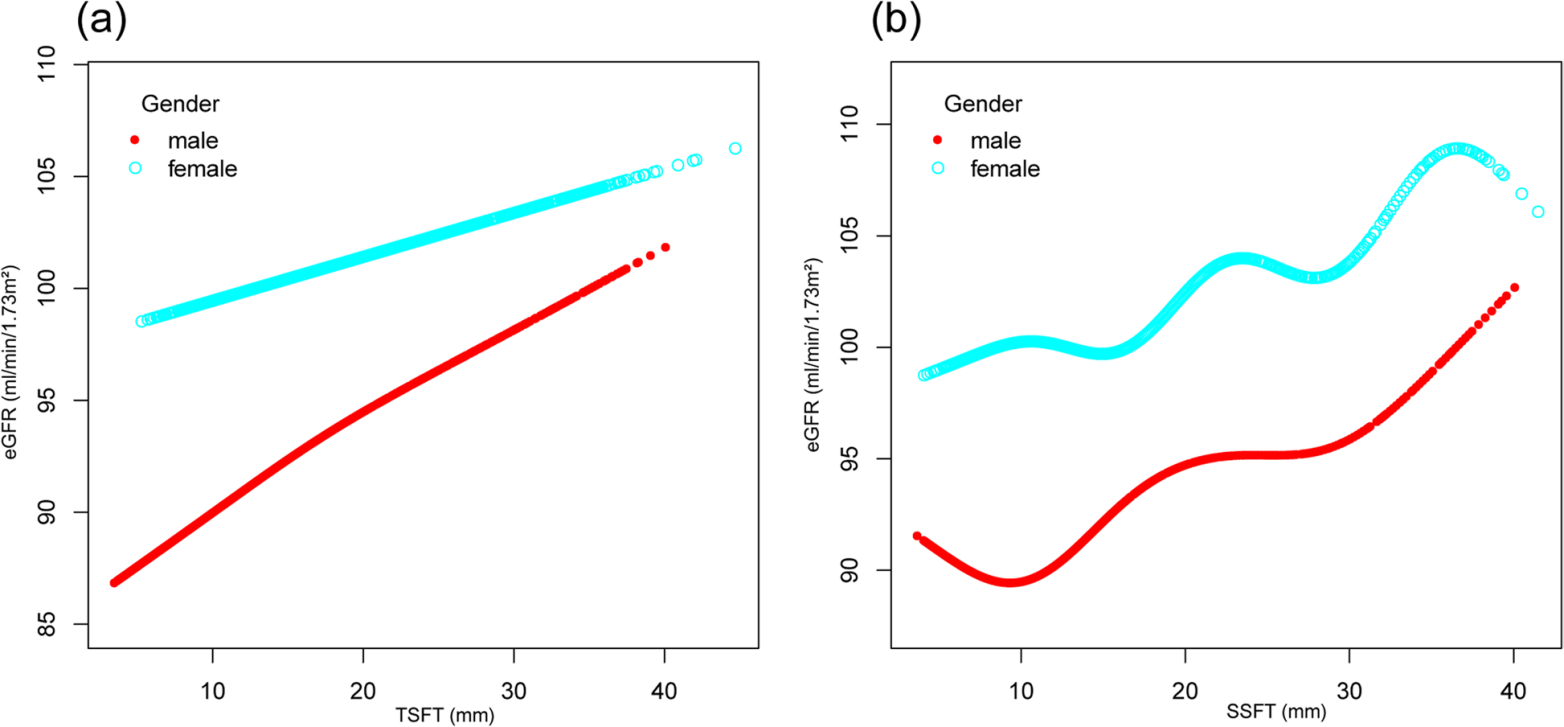
Fitting smooth curve between skinfold and eGFR. **a** The association between TSFT and eGFR in adolescent. **b** The association between SSFT and eGFR in adolescent. Each point or circle represents a sample. Age, race/ethnicity, standing height, annual family income, blood urea nitrogen and uric acid were adjusted. Abbreviations: TSFT, triceps skinfold thickness; SSFT, subscapular skinfold thickness

To further investigate the linear relationship between skinfold thickness and eGFR in adolescents, we used a GAM and a fitted smoothing curve (Fig. 1). The results showed that there was a linear relationship between TSFT and eGFR in boys (β = 0.389, *P* < 0.001) and girls (β = 0.159, *P* = 0.0003): eGFR increased by 0.389 mL/min/1.73 m^2^ in boys and 0.159 mL/min/1.73 m^2^ in girls for each increase 1 mm of TSFT. The relationship between SSFT and eGFR was curvilinear.

## Discussion

Overweight and obesity are well-known risk factors for renal function loss. A BMI of more than 25 kg/m^2^ increases the long-term risk of end-stage renal disease by two to three times [19], but the relationship between obesity and glomerular filtration rate is unclear. Therefore, we examined the association of eGFR and glomerular hyperfiltration with TSFT and SSFT derived from the NHANES database in adolescents from 2001 to 2010.

We found that in both boys and girls, the younger the age and the lower the weight and BMI, the less likely eGFR was to increase (*P* < 0.05). Further threshold effect analysis showed that weight and BMI greater than 66.8 and 20.59, respectively, were positively correlated with eGFR (Supplementary Table 1).

The pathogenesis of glomerular hyperfiltration in obese patients is quite complex and has not been fully understood. In patients with diabetes and hypertension, high BMI has been shown to be one of the major factors for glomerular hyperfiltration [20]. An elevated BMI also contributed to the progression of chronic kidney disease in patients without high blood pressure or diabetes [21]. Interestingly, weight loss resulted in reduced hyperfiltration in severely obese patients [22]. Some studies have reported that glomerular hyperfiltration begins to occur when the body begins to accumulate excess fat [23]; this finding is consistent with the positive correlation noted between TSFT, SSFT and eGFR in adolescents in our study. Obesity-related kidney damage is thought to be caused by factors such as hyperlipidemia, increased oxidative stress, increased salt intake, and sympathetic nervous system activation [24]. Oxidative stress, secondary to increased obesity, is also thought to be a risk factor of hyperfiltration. Increased oxidation of low-density lipoproteins, observed in obese patients, stimulates angiotensin II synthesis, which in turn promotes glomerular fibrosis and chronic kidney disease caused by inflammatory cytokines [25]. Skinfold thickness reflects excess body fat content, and excess body fat may lead to increased glomerular filtration rate through previous pathways.

The observed association between TSFT, SSFT and eGFR in adolescent boys, but no significant association between TSFT, SSFT and glomerular hyperfiltration in adolescent girls, may the result of lower glomerular hyperfiltration occurrence in girls. A study assessing glomerular hyperfiltration in humanized sickle cell mice showed that increased age and male sex were risk factors for the development of glomerular hyperfiltration [26]; this is consistent with the finding of our study. We observed that TSFT, SSFT were positively correlated with eGFR and glomerular hyperfiltration in boys, and the increase of eGFR in boys was greater than that in girls when TSFT increased by 1 mm. The gender difference in the correlation between TSFT and glomerular hyperfiltration may be related to the protective effect of females without diabetic nephropathy [27], and the specific physiological mechanism may be related to the reduction of vascular responsiveness to renin angiotensin system activation by estrogen [28]. Studies in rat models have shown that estrogen attenuates angiotensin II-induced blood perfusion [29].

To the best of our knowledge, this is the first study to identify TSFT and SSFT as an indicator of glomerular hyperfiltration in adolescents and to provide theoretical support for the association between the incidence of glomerular hyperfiltration and BMI. Moreover, it is simpler and more convenient to determine skinfold thickness than it is to determine the eGFR using the Schwartz equation.

Our study has some limitations. First, this study is a cross sectional analysis, so it provides only weak evidence of associations between independent variables and outcomes. As the study only included US adolescents, these results may not apply to other ethnic groups. Second, the measurement error of skinfold thickness is significant and unavoidable [30]. In NHANES, skinfold thickness was measured by highly trained medical personnel, so skinfold thickness can only be used as a preliminary indicator of the incidence of glomerular hyperfiltration in clinical practice. Third, although, eGFR obtained by the Schwartz equation has been used in previous studies on glomerular hyperfiltration, it does have the potential to overestimate eGFR. To avoid bias, we applied the new European Kidney Function Consortium (EKFC) equation [31] to further calculate eGFR, and the correlation between skinfold thickness and eGFR-EKFC was consistent with the results of this study (Supplementary Table 2).

## Conclusion

TSFT and SSFT are positively correlated with eGFR and glomerular hyperfiltration in US male adolescents. Furthermore, in all adolescents, there is a linear relationship between TSFT and eGFR. This provides theoretical support for the association between skinfold thicknesses and the incidence of glomerular hyperfiltration.

## Supplementary Information

**Additional file 1: Table 1.** Threshold effect analysis between the weight, BMI and eGFR.

**Additional file2: Table 2.** Multivariate regression analysis of the correlations between the eGFR-EKFC and skinfold by gender.

## Data Availability

Data can be downloaded from the ‘NHANES’ database (https://www.cdc.gov/nchs/nhanes/index.htm).

## Abbreviations

BMI: Body mass index
BUN: Blood urea nitrogen
CI: Confidence interval
eGFR: Estimated glomerular filtration rate
OR: Odds ratio
Scr: Serum creatinine
SSFT: Subscapular skinfold thickness
TSFT: Triceps skinfold thickness
