# Supplementary material for: The association between skinfold thicknesses and estimated glomerular filtration rate in adolescents: a cross-sectional study
Source: BMC Nephrol. 2022 Mar 5;23:96. doi: 10.1186/s12882-022-02709-7 (PMC8897831; doi:10.1186/s12882-022-02709-7)
Supplement: Supplementary file 1 — Additional file 1: Table 1. Threshold effect analysis between the weight, BMI and eGFR. [file 12882_2022_2709_MOESM1_ESM.doc]

### Supplementary Table 1. Threshold effect analysis between the weight, BMI and eGFR.

|  | weight(kg) | body mass index(kg/m2) |
| --- | --- | --- |
| Kink(K) | 66.8 | 20.59 |
| < K segment effect 1 | -0.095 (-0.145, -0.045) 0.0002 | -1.184 (-1.500, -0.867) <0.0001 |
| > K segment effect 2 | 0.206 (0.164, 0.248) <0.0001 | 0.507 (0.404, 0.610) <0.0001 |
| - 2＆1 variability of effectiveness | 0.301 (0.226, 0.375) <0.0001 | 1.690 (1.324, 2.056) <0.0001 |
| Predicted value of the equation at the break point | 90.293 (89.542, 91.044) | 93.071 (92.365, 93.777) |
| Logarithmic likelihood ratio test | <0.001 | <0.001 |

Above are adjusted for age, race, blood urea nitrogen, and uric acid.
