# Supplementary material for: The association between skinfold thicknesses and estimated glomerular filtration rate in adolescents: a cross-sectional study
Source: BMC Nephrol. 2022 Mar 5;23:96. doi: 10.1186/s12882-022-02709-7 (PMC8897831; doi:10.1186/s12882-022-02709-7)
Supplement: Supplementary file 2 — Additional file2: Table 2. Multivariate regression analysis of the correlations between the eGFR-EKFC and skinfold by gender. [file 12882_2022_2709_MOESM2_ESM.doc]

### Supplementary Table 2. Multivariate regression analysis of the correlations between the eGFR-EKFC and skinfold by gender.

| Exposure | Effect size OR (95%CI), P-value | | |
| --- | --- | --- | --- |
|  | Crude model | Minimally adjusted model | Fully adjusted model |
| Male |  |  |  |
| TSFT(mm) | 0.267 (0.202, 0.333) <0.00001 | 0.190 (0.130, 0.250) <0.00001 | 0.304 (0.245, 0.364) <0.00001 |
| SSFT(mm) | 0.013 (-0.055, 0.082) 0.70277 | 0.102 (0.039, 0.165) 0.00142 | 0.247 (0.184, 0.310) <0.00001 |
| Female |  |  |  |
| TSFT(mm) | -0.044 (-0.118, 0.030) 0.24526 | 0.051 (-0.016, 0.117) 0.13736 | 0.148 (0.081, 0.215) 0.00002 |
| SSFT(mm) | -0.007 (-0.077, 0.062) 0.83536 | 0.088 (0.025, 0.151) 0.00598 | 0.194 (0.130, 0.259) <0.00001 |
| Total |  |  |  |
| TSFT(mm) | 0.126 (0.077, 0.176) <0.00001 | 0.105 (0.061, 0.150) <0.00001 | 0.188 (0.144, 0.233) <0.00001 |
| SSFT(mm) | 0.003 (-0.046, 0.052) 0.90456 | 0.092 (0.047, 0.136) 0.00005 | 0.202 (0.157, 0.247) <0.00001 |

Abbreviations: eGFR-EKFC, estimated glomerular filtration rate by new European Kidney Function Consortium; TSFT, triceps skinfold thickness; SSFT, subscapular skinfold thickness.

Note: Crude model: We did not adjust any covariants. Minimally adjusted model: We adjusted age, race, and standing height. Fully adjusted model: We adjusted age, standing height, race, family income, blood urea nitrogen and uric acid variables.

**The new European Kidney Function Consortium equation (EKFC):**

| Age | Scr/Q | Equation |
| --- | --- | --- |
| 2-40 y | <1 | 107.3×(Scr/Q)^-0.322^ |
|  | ≥1 | 107.3×(Scr/Q)^-1.132^ |
| >40 y | <1 | 107.3×(Scr/Q)^-0.322^×0.990^(Age-40)^ |
|  | ≥1 | 107.3×(Scr/Q)^-1.132^×0.990^(Age-40)^ |

Q values

For ages 2-25 y:

Males:

ln(Q)=3.200 + 0.259 × Age - 0.543 × ln(Age) - 0.00763 × Age^2^ + 0.0000790 × Age^3^

Females:

ln(Q)=3.080 + 0.177 × Age - 0.223 × ln(Age) - 0.00596 × Age^2^ + 0.0000686 × Age^3^

For ages >25 y:

Males: Q=80 µmol/L (0.90 mg/dL)

Females:Q=62 µmol/L (0.70 mg/dL)

Q values (in µmol/L or mg/dL) correspond to the median Scr values for the age- and sex-specific populations. Scr, serum creatinine.
